# Multi-Party Verifiably Collaborative Encryption for Biomedical Signals via Singular Spectrum Analysis-Based Chaotic Filter Bank Networks

**DOI:** 10.3390/s25123823

**Published:** 2025-06-19

**Authors:** Xiwen Zhang, Jianfeng He, Bingo Wing-Kuen Ling

**Affiliations:** School of Information Engineering, Guangdong University of Technology, Guangzhou 511006, China; 2112303041@mail2.gdut.edu.cn (X.Z.); 2112303129@mail2.gdut.edu.cn (J.H.)

**Keywords:** biomedical signal encryption, chaotic filter bank networks, singular spectrum analysis, multi-party collaborative encryption, verifiable decryption

## Abstract

This paper proposes a multi-party verifiably collaborative system for encrypting the nonlinear and the non-stationary biomedical signals captured by biomedical sensors via the singular spectrum analysis (SSA)-based chaotic networks. In particular, the raw signals are first decomposed into the multiple components by the SSA. Then, these decomposed components are fed into the chaotic filter bank networks for performing the encryption. To perform the multi-party verifiably collaborative encryption, the window length of the SSA and the total number of the layers in the chaotic network are flexibly designed to match the total number of the collaborators. The computer numerical simulation results show that our proposed system achieves a good encryption performance.

## 1. Introduction

The secure transmission of biosensor signals presents unique challenges in privacy-preserving telemedicine. Wearable and implantable medical devices generate continuous streams of electrophysiological data such as EEG and PPG that contain sensitive health biomarkers [1,2,3]. These IoT-enabled biosensors operate under distinctive security constraints, where ultra-low-power operation limits complex cryptographic processing capabilities, while wireless transmission exposes time-series data to potential interception. The biomedical community consequently requires robust encryption systems that can address these challenges while maintaining the integrity and privacy of biosensor signals.

Secure biomedical encryption requires cryptosystems that fulfill Shannon’s confusion–diffusion criteria [4]. Chaotic maps offer inherent advantages through their sensitive dependence on initial conditions and nonlinear transformation properties. However, conventional chaotic systems, including logistic maps [5,6,7] and cat maps [8], face practical limitations due to their constrained key spaces. Current frequency-domain approaches, including chaotic filter banks [5] and EMD-based methods [7], introduce additional vulnerabilities because their explicit frequency-domain representations create identifiable attack surfaces.

The increasing demand for of secure biosensor data sharing has necessitated the evolution of multi-party collaborative frameworks as viable alternatives to conventional trusted third-party architectures. These distributed systems enforce strict access control through cryptographic consensus mechanisms. Secure Multi-Party Computation (SMPC) [9,10,11,12] enables this privacy-preserving distributed computing paradigm through advanced cryptographic techniques. Fully Homomorphic Encryption (FHE) [13,14] has emerged as a promising solution for cross-institutional collaboration, as it maintains end-to-end privacy by supporting computations on encrypted data without requiring decryption [15]. However, the approach faced practical barriers, including substantial computational overhead and ciphertext expansion, that strain bandwidth-constrained biosensor networks. Cloud-based acceleration has significantly improved homomorphic encryption performance through hardware optimization [16,17]. While effectively addressing computational bottlenecks, these solutions require specialized infrastructure and still face the inherent challenge of ciphertext expansion in FHE systems.

Distributed storage architectures offer potential solutions to these challenges by spreading ciphertext storage across multiple parties, thereby reducing individual storage burdens. Blockchain systems [18,19] typically use AES encryption [20], which restricts decryption to single key-holders, preventing direct multi-party decryption without key-sharing protocols. Alternative cryptographic designs based on polynomial interpolation [21] has shown promise for healthcare applications [22], though they introduce their own computational complexities during decryption that scale quadratically with the number of reconstruction shares. Moreover, SMPC encounters communication overhead challenges in real-time environments.

The verifiability of cryptographic operations represents another critical requirement for effective multi-party collaboration in medical contexts. Traditional secret-sharing schemes lack built-in mechanisms for verifying decryption accuracy, creating potential vulnerabilities if ciphertexts are intentionally modified during transmission. Recent advances have proposed verifiable schemes based on polynomial interpolation [23] and demonstrated their application in multi-cloud environments [2]. Nevertheless, verifiable decryption mechanisms tailored for chaotic cryptosystems and nonlinear signal processing remain underdeveloped in current research.

To address these challenges, this paper proposes a novel multi-party verifiably collaborative system for biosensor signal encryption based on SSA and chaotic filter bank networks. Our approach implements confusion through SSA decomposition followed by polyphase filtering and multi-layer chaotic network processing. The diffusion phase leverages the dynamic properties of chaotic systems where initial conditions propagate throughout the network architecture. The system’s unique architecture inherently enables multi-party encryption with built-in verification capabilities. The remainder of this paper details our cryptosystem design, security analysis, and computational complexity, presents comprehensive simulation results, and discusses the implications of our findings for future research in secure medical data transmission.

## 2. Proposed Cryptosystem

**Figure 1:**
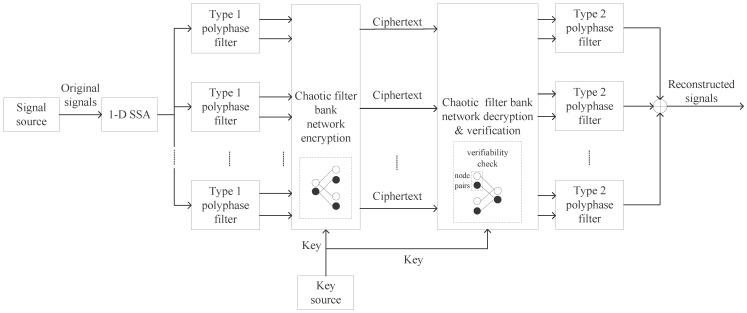
The framework of the proposed encryption and decryption system.

Figure 1 illustrates the end-to-end framework of the proposed encryption and decryption system. The decryption process is implemented as the inverse operation of the encryption algorithm. Specifically, the verification ensures data integrity by requiring consensus between two node pairs’ decryption before accepting any output of the chaotic filter bank network.

### 2.1. Decomposition and Reconstruction of Biosensor Signals Based on SSA and Haar Filter Bank

**Figure 2:**
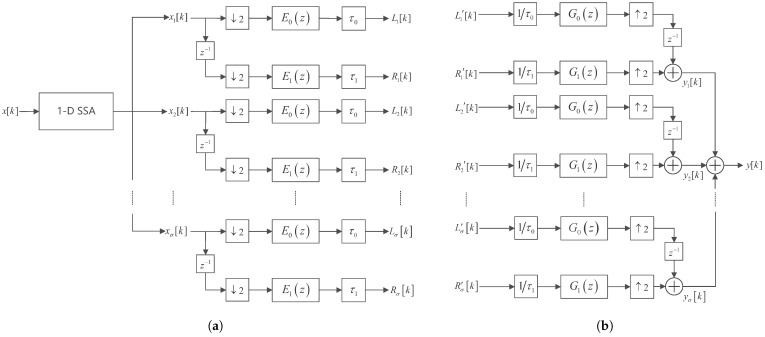
(**a**) The analysis and (**b**) synthesis system of the proposed method.

Figure 2a,b shows the analysis and synthesis system of the proposed method, respectively. Here, biosensor signals are decomposed by both SSA and filter bank. Let x[k] be a biomedical signal. Let $n_{1}$ be its length. Let σ be the total number of the nonzero singular values of the trajectory matrix X generated by x[k], that is, $X=\sum_{i=1}^{\sigma }X_{i}$, where $X_{i}$ is the component matrix. These component matrices can be divided into *h* disjoint subsets, that is $X=\sum_{i=1}^{h}X_{I_{i}}$, where $X_{I_{i}}$ is the ith grouped matrix. For simplicity, let h=σ. For each $X_{I_{i}}$, de-Hankelization is performed to form $x_{i}\left[k\right]$ for i=1,2,…,σ, where $x_{i}\left[k\right]$ is the ith singular spectral decomposition components of $x\left[k\right]$, that is, $x\left[k\right]=\sum_{i=1}^{\sigma }x_{i}\left[k\right]$.

Each SSA component is processed using Haar filter banks. Let $L_{i}\left[k\right]$ and $R_{i}\left[k\right]$ be the first and second subband signal of the ith SSA component decomposed by the Haar analysis filter bank [24]; ${L_{i}}^{\prime }\left[k\right]$ and ${R_{i}}^{\prime }\left[k\right]$ be the corresponding decrypted subband signals for synthesis; ↓2 and ↑2 be the 2-fold downsampler and upsampler; $e_{i}\left[k\right]$ and $g_{i}\left[k\right]$ for i=0,1 be the impulse responses of the Haar analysis and synthesis filters; and $\tau_{0}$ and $\tau_{1}$ be the gains multiplied to the low-frequency and high-frequency subband channel, respectively. Let $z^{-1}$ be a unit delay element. The output of the Haar synthesis filter bank for reconstructing the ith SSA component is denoted as $y_{i}\left[k\right]$, and the final reconstructed signal is $y\left[k\right]=\sum_{i=1}^{\sigma }y_{i}\left[k\right]$.

The various signals in Figure 2 can be expressed as follows:

(1) $$L_{i}\left[k\right]=\tau_{0}\sum_{\forall m}x_{i}\left[2k\right]e_{0}\left[k-m\right],$$

(2) $$R_{i}\left[k\right]=\tau_{1}\sum_{\forall m}x_{i}\left[2k-1\right]e_{1}\left[k-m\right].$$

(3) $$y_{i}\left[k\right]=\frac{1}{\tau_{0}}\sum_{\forall m}{L_{i}}^{\prime }\left[2k-1\right]g_{0}\left[k-m\right]+\frac{1}{\tau_{1}}\sum_{\forall m}{R_{i}}^{\prime }\left[2k\right]g_{1}\left[k-m\right].$$

The type 1 polyphase matrix of the Haar analysis filter bank [5,24] and the impulse response of the Haar mother wavelet [5] are

(4) $$E\left(z\right)=\left[\begin{array}{cc} 1 & 1\\ 1 & -1 \end{array}\right]$$

and

(5) $$\psi \left(u\right)=\left\{\begin{array}{cc} 1 & 0<u<0.5\\ -1 & 0.5<u<1\\ 0 & \;\mathrm{otherwise}\; \end{array}.\right.$$

Let $G\left(z\right)$ be the type 2 polyphase matrix of the Haar synthesis filter bank. Let c=1 and $m_{0}=0$ be the gain and the delay introduced by the Haar filter bank, respectively. Since the Haar filter bank achieves exact perfect reconstruction, we have $E\left(z\right)G\left(z\right)=cz^{-m_{0}}I$, where I is an identity matrix [25].

### 2.2. Dynamics of Nodes in Various Layers of Analysis Network and Synthesis Network

Let γ be the total number of layers in the analysis network. Let $\sigma_{l}$ for l=1,2,…,γ be the total number of node pairs in the lth layer of the analysis network. Let $F_{j,i,l}\left(\cdot \right)$, for j=0,1, for $i=1,2,\ldots ,\sigma_{l}$, and for l=1,2,…,γ, be the chaotic functions [26] of the jth subband in the ith node of the lth layer of the analysis network. Let $\lambda_{j,i,l}$ be the parameter in $F_{j,i,l}\left(\cdot \right)$. Let $a_{j,i,l}\left[k\right]$, $v_{j,i,l}\left[k\right]$, and $b_{j,i,l}\left[k\right]$ be the state variable, the input, and the output of $F_{j,i,l}\left(\cdot \right)$, respectively. Then, the dynamics of $F_{j,i,l}\left(\cdot \right)$ are

(6) $$a_{j,i,l}\left[k+1\right]=\lambda_{j,i,l}a_{j,i,l}\left[k\right]\left(1-a_{j,i,l}\left[k\right]\right)$$

and

(7) $$b_{j,i,l}\left[k\right]=a_{j,i,l}\left[k\right]+v_{j,i,l}\left[k\right].$$

The inputs of the (i,l)th analysis node are denoted as $L_{i,l}\left[k\right]$ and $R_{i,l}\left[k\right]$, and the corresponding outputs are denoted as $P_{i,l}\left[k\right]$ and $Q_{i,l}\left[k\right]$. Then, the dynamics are

(8) $$P_{i,l}\left[k\right]=L_{i,l}\left[k\right]+F_{0,i,l}\left(R_{i,l}\left[k\right],a_{0,i,l}\left[k\right]\right)$$

and

(9) $$Q_{i,l}\left[k\right]=R_{i,l}\left[k\right]-F_{1,i,l}\left(L_{i,l}\left[k\right],a_{1,i,l}\left[k\right]\right).$$

**Figure 3:**
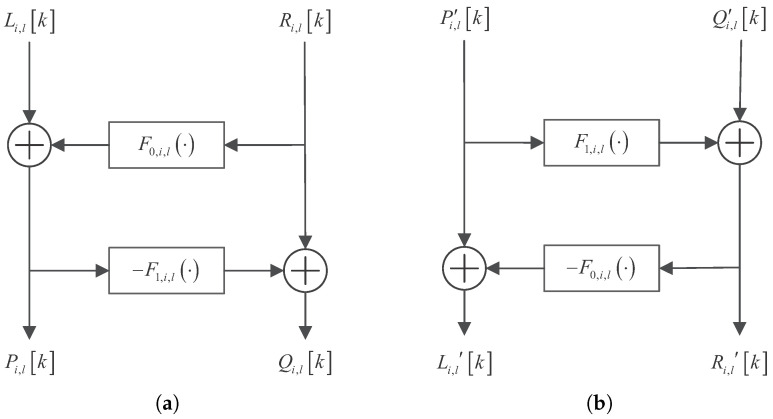
The structure of the (i,l)th node of the (**a**) analysis and (**b**) synthesis network.

The structure of the (i,l)th node of the analysis network is shown in Figure 3a.

Similarly, the inputs of the (i,l)th synthesis node are denoted as ${P^{\prime }}_{i,l}\left[k\right]$ and ${Q^{\prime }}_{i,l}\left[k\right]$, and the corresponding outputs are denoted as ${L_{i,l}}^{\prime }\left[k\right]$ and ${R_{i,l}}^{\prime }\left[k\right]$. Then, the dynamics are

(10) $${R_{i,l}}^{\prime }\left[k\right]={Q^{\prime }}_{i,l}\left[k\right]+F_{1,i,l}\left({P^{\prime }}_{i,l}\left[k\right],a_{1,i,l}\left[k\right]\right)$$

and

(11) $${L_{i,l}}^{\prime }\left[k\right]=P_{i,l}\left[k\right]-F_{0,i,l}\left({Q_{i,l}}^{\prime }\left[k\right],a_{0,i,l}\left[k\right]\right).$$

The structure of the (i,l)th node of the synthesis network is shown in Figure 3b.

### 2.3. Multi-Layer Chaotic Network for Performing Multi-Party Verifiable Collaborative Encryption

**Figure 4:**
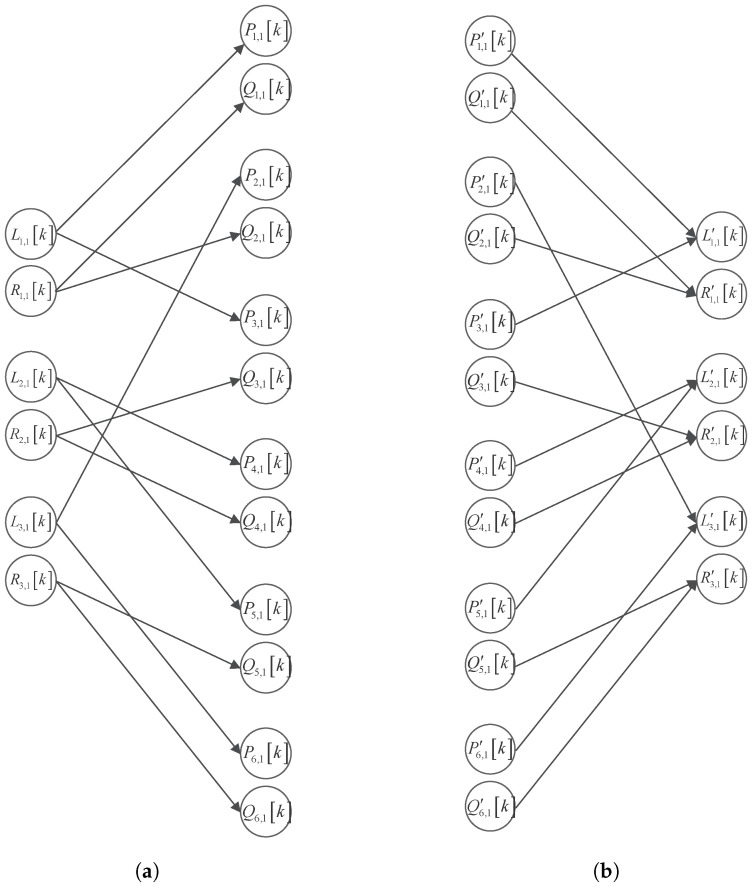
An example ($\sigma_{1}=3,\sigma_{2}=6$) of the input–output connections of the first layer in (**a**) analysis and (**b**) synthesis chaotic filter bank network.

This paper proposes a multi-layer chaotic filter bank network for performing a multi-party verifiable collaborative encryption. Take the first layer network as an example. $L_{i,1}\left[k\right]$ and $R_{i,1}\left[k\right]$ are randomly chosen as the inputs of the chaotic filter bank to obtain $P_{i,1}\left[k\right]$ and $Q_{i,1}\left[k\right]$, respectively. Also, $L_{i,1}\left[k\right]$ and $R_{i,1}\left[k\right]$ are taken twice for each *i*. Thus, $\sigma_{2}=2\sigma_{1}=2\sigma$. Let $A_{l}$ for l=1,2,…,γ be the matrix governing the connection between $L_{i,l}\left[k\right]$ and $P_{i,l}\left[k\right]$, as well as $B_{l}$ for l=1,2,…,γ be the matrix governing the connection between $R_{i,l}\left[k\right]$ and $Q_{i,l}\left[k\right]$. Figure 4a shows an example of the first-layer analysis network’s input–output connections. Setting $\sigma_{1}=3$, we observe that

(12) $$A_{l}\in R^{\sigma_{2}\times \sigma_{1}}={\left(\alpha_{ab}\right)}_{\sigma_{2}\times \sigma_{1}}=\left[\begin{array}{ccc} 1 & 0 & 0\\ 0 & 0 & 1\\ 1 & 0 & 0\\ 0 & 1 & 0\\ 0 & 1 & 0\\ 0 & 0 & 1 \end{array}\right]$$

and

(13) $$B_{l}\in R^{\sigma_{2}\times \sigma_{1}}={\left(\beta_{ab}\right)}_{\sigma_{2}\times \sigma_{1}}=\left[\begin{array}{ccc} 1 & 0 & 0\\ 1 & 0 & 0\\ 0 & 1 & 0\\ 0 & 1 & 0\\ 0 & 0 & 1\\ 0 & 0 & 1 \end{array}\right],$$

where $\alpha_{ij}=1$ if $P_{a,1}\left[k\right]$ is the output when $L_{b,1}\left[k\right]$ is the input; otherwise, $\alpha_{ij}=0$. Likewise, $\beta_{ij}=1$ if $Q_{a,1}\left[k\right]$ is the output when $R_{b,1}\left[k\right]$ is the input; otherwise, $\beta_{ij}=0$.

The analysis network with total γ layers generates $\sigma 2^{\gamma }$ distinct output signals, which are distributed as unique ciphertexts among $\sigma 2^{\gamma }$ recipients. Each recipient thus possesses an exclusive portion of the signal’s complete information.

To decrypt the complete signal, all $\sigma 2^{\gamma }$ distributed ciphertexts must be collected and processed through the synthesis network (Figure 4b), which implements the inverse transformation using matrices $A_{l}^{T}$ and $B_{l}^{T}$. The system features redundant reconstruction paths, where different inputs (e.g., $P_{1,1}^{\prime }\left[k\right]$ and $Q_{1,1}^{\prime }\left[k\right]$, or $P_{3,1}^{\prime }\left[k\right]$ and $Q_{2,1}^{\prime }\left[k\right]$) can yield identical outputs ($L_{1,1}^{\prime }\left[k\right]$ and $R_{1,1}^{\prime }\left[k\right]$), enabling a built-in verification mechanism. By comparing reconstruction results from independent ciphertexts, the system can detect tampering—successful verification occurs when both paths agree—while discrepancies identify potentially tampered ciphertexts through an analysis of $A_{\gamma }^{T}$ and $B_{\gamma }^{T}$. This design provides both tamper evidence and fault localization while ensuring correct decryption when all parties provide authentic ciphertexts.

## 3. Security Analysis and Computational Complexity

### 3.1. Dynamic Characteristics of the Logistic Map

The nonlinear dynamical system in (6) exhibits chaotic behavior with state variables $a_{j,i,l}\left[k\right]\in A$, where A⊆(0,1) is the state space, and control parameters $\lambda_{j,i,l}\in B$, where $B\subseteq \left[0,4\right]$ is the parameter space.

The Lyapunov exponent ϕ for $a_{j,i,l}\left[k+1\right]=F_{j,i,l}\left(a_{j,i,l}\left[k\right]\right)$ is

(14) $$\phi \left(a_{j,i,l}\left[0\right]\right)=\lim_{T\to \infty }\;\frac{1}{T}\sum_{m=0}^{T-1}\ln\left|F^{\prime }\left(a_{j,i,l}\left[m\right]\right)\right|,\;a_{j,i,l}\left[0\right]\in A.$$

**Figure 5:**
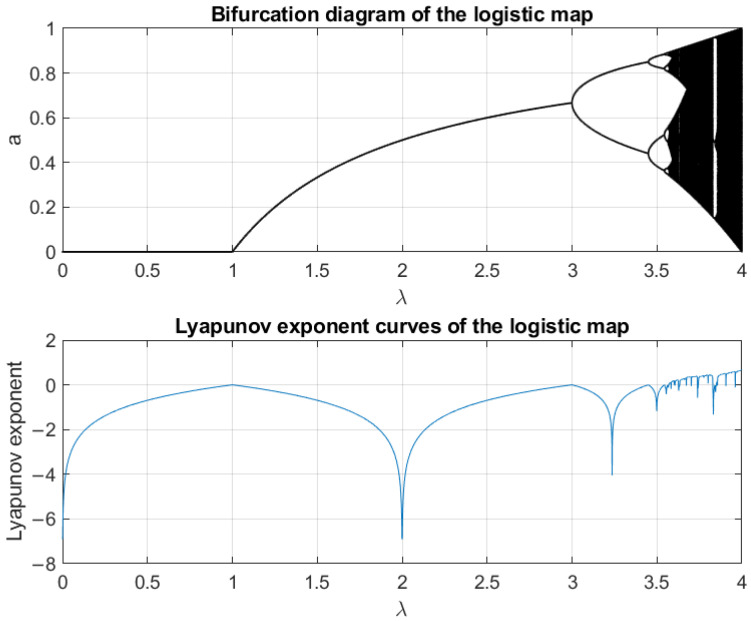
Bifurcation diagram and Lyapunov exponent curve of the logistic map.

Figure 5 shows the bifurcation and Lyapunov diagrams. For $\lambda_{j,i,l}\in \left[0,1\right]$, the system has a single fixed point. For $\lambda_{j,i,l}\in \left(1,3\right]$, the system has a stable cycle. For $\lambda_{j,i,l}\in \left(3,3.57\right)$, the system is periodic doubling. Chaos begins to appear when $\lambda_{j,i,l}$ reaches Feigenbaum’s universal constant $\lambda_{\infty }$. Here, $\lambda_{\infty }\approx 3.57$. When $\lambda_{j,i,l}>4$, the system is not stable. Thus, the chaotic region is defined as $\lambda_{j,i,l}\in \left[3.57,4\right]$.

### 3.2. Keyspace Analysis

The key consists of $\lambda_{j,i,l}$, $a_{j,i,l}\left[0\right]$, $A_{l}^{T}$, and $B_{l}^{T}$. Let υ be the window length of SSA. Note that, for typical biological signals, the trajectory matrix X is generally a full-rank matrix, meaning that υ=σ. Let $m_{1}$ and $m_{2}$ be the storage precision of $a_{j,i,l}\left[0\right]$ and $\lambda_{j,i,l}$, respectively. The size of the keyspace is

(15) ∏p=1γ22p×2υm1×22p×2υm2∏q=12γ2∏t=0qυ−12qυ−2t2,

where nk=n!k!(n−k)! for 0≤k≤n, and (·)! denotes factorial. Compared to the encryption method in [5] with a keyspace of $2^{2m_{1}}\times 2^{2m_{2}}$, the keyspace of the proposed encryption system is greatly improved.

### 3.3. Algorithmic Complexity Analysis

The system’s computational load arises from SSA (SVD-dominated, $O(\upsilon n_{1}^{2})$) and the chaotic filter bank network ($O(2^{\gamma +1}\upsilon n_{1})$). Decryption requires only $O(2^{\gamma +1}\upsilon n_{1})$ operations, as it bypasses SVD. For biosignals where $n_{1}\gg \upsilon$, the SVD term governs total encryption cost. To address the computational bottleneck of SSA when processing long signals ($n_{1}>10^{3}$), a segmented processing approach can be employed. Apart from that, the chaotic network’s $O(2^{\gamma +1}\upsilon n_{1})$ cost grows exponentially with γ, but remains tractable for small γ (e.g., γ≤4).

The ciphertext expansion ratio quantifies the size increase from plaintext to ciphertext in an encryption scheme. This metric directly determines the feasibility of the encryption scheme in real-time biosignal processing. The ciphertext expansion ratio of our proposed method is $\sigma 2^{\gamma }$, which grows exponentially with the number of design layers γ. However, a five-layer design already offers excellent encryption performance, allowing up to 32σ parties to access the encrypted signal.

## 4. Computer Numerical Simulation Results

The real-time monitoring of electroencephalogram (EEG) signals plays a critical role in diagnosing and managing neurological disorders, while photoplethysmogram (PPG) signals provide essential cardiovascular information through heart rate monitoring. Both EEG and PPG exhibit inherent nonlinearity and non-stationarity, making them ideal test cases for evaluating our proposed encryption system. In this study, experimentally acquired EEG and PPG signals are encrypted to demonstrate the system’s effectiveness and flexibility in achieving multi-party cooperative and verifiable encryption for complex biomedical signals. The performance is rigorously compared with the logistic chaotic filter bank encryption method reported in [5] and other mainstream encryption approaches. In the experiments, $\tau_{0}=0.001$, $\tau_{1}=0.004$, $a_{0,i,l}\left[0\right]=0.7$, and $a_{1,i,l}\left[0\right]=0.9$. In [5], $\lambda_{0}=3.98$ and $\lambda_{1}=4.00$. In encrypting based on SSA and the proposed chaotic network, $\lambda_{0,i,l}=3.98$ and $\lambda_{1,i,l}=4.00$. To establish a rigorous baseline evaluation of our method’s core functionality, we implemented a minimal test configuration featuring SSA with a conservative window length (υ≤5) and a simplified network architecture limited to three layers.

### 4.1. Data Acquisition

**Figure 6:**
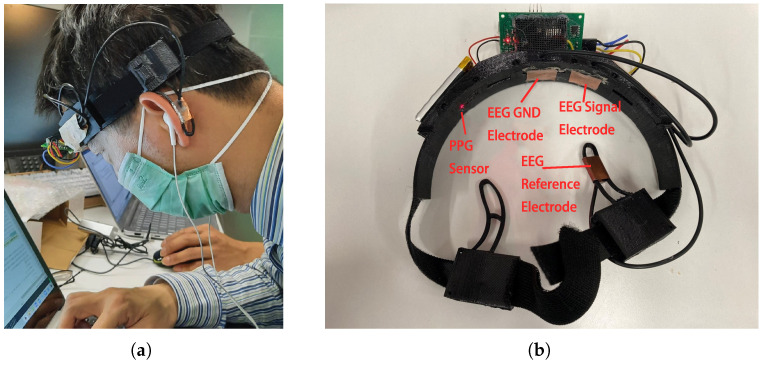
The wearable headband’s (**a**) deployment in actual measurement scenarios and (**b**) design for EEG and PPG acquisition.

The study employed a custom wearable headband that integrated dual-modality biosignal acquisition. The wearable headband’s design and its deployment in actual measurement scenarios are illustrated in Figure 6, demonstrating the practical usage conditions during physiological signal acquisition. For EEG signal acquisition, we implemented the NeuroSky ThinkGear ASIC Module (TGAM), a specialized biosignal acquisition integrated circuit configured with a 512 Hz sampling frequency. The electrode montage follows standard electrophysiological recording protocols, comprising three essential contact points: the left earlobe serving as reference electrode, the left forehead as EEG signal electrode, and the mid-forehead as GND electrode. Simultaneously, the device incorporates a reflective photoplethysmography sensor positioned at the right forehead to acquire signals at a sampling frequency of 100 Hz.

### 4.2. Reconstruction Error

**Figure 7:**
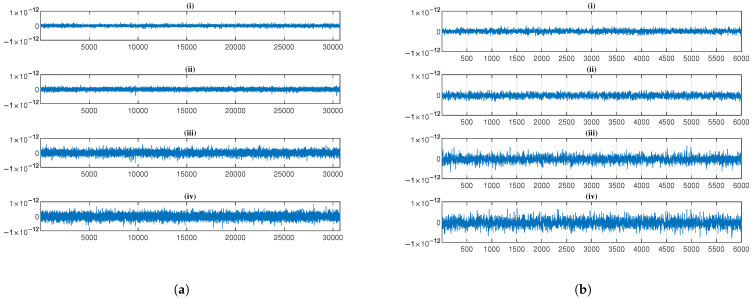
Reconstruction error of the 1 min (**a**) EEG and (**b**) PPG across four encryption methods, with each subplot (i)–(iv) representing (i) the logistic chaotic filter bank approach from [5], (ii) our SSA with 1-layer chaotic network, (iii) SSA with 2-layer network, and (iv) SSA with 3-layer network.

Figure 7 presents a comparative analysis of decryption reconstruction errors for Figure 7a EEG and Figure 7b PPG across four encryption methods, with each subplot (i)–(iv) representing (i) the logistic chaotic filter bank approach from [5], (ii) our SSA with 1-layer chaotic network, (iii) SSA with 2-layer network, and (iv) SSA with 3-layer network. The results demonstrate consistently minimal reconstruction errors across all methods.

Mean square error (MSE) is used to test the effectiveness of reconstruction accuracy after signal encryption and is defined as

(16) $$MSE\left(X,Y\right)=\frac{\sum_{i=1}^{N}{\left(x_{i}-y_{i}\right)}^{2}}{N},$$

where *X* and *Y* are the original signal and the decrypted reconstruction signals, respectively, and each of the two signals has *N* scalar observations.

The percentage root-mean-square difference (PRD) is used to assess the degree of distortion in the encryption system and is defined as

(17) $$PRD\left(X,Y\right)=100\times \sqrt{\frac{\sum_{i=1}^{N}{\left(x_{i}-y_{i}\right)}^{2}}{\sum_{i=1}^{N}{x_{i}}^{2}}},$$

and the physical meaning of the variables here is the same as in (16).

Table 1 presents the reconstruction performance metrics (MSE and PRD) for the four evaluated encryption methods. Theoretically, these methods are capable of perfect signal reconstruction, which is substantiated by the experimental results showing extremely low error magnitudes: MSE values on the order of $10^{-28}$ to $10^{-27}$ and PRD values of $10^{-14}$ to $10^{-13}$ for both EEG and PPG signals. These negligible errors primarily stem from inherent numerical computation limitations rather than methodological deficiencies. While the approach in [5] demonstrates marginally smaller errors, all methods maintain reconstruction fidelity well within acceptable thresholds.

### 4.3. Signal Processing and Encryption Visualization

**Figure 8:**
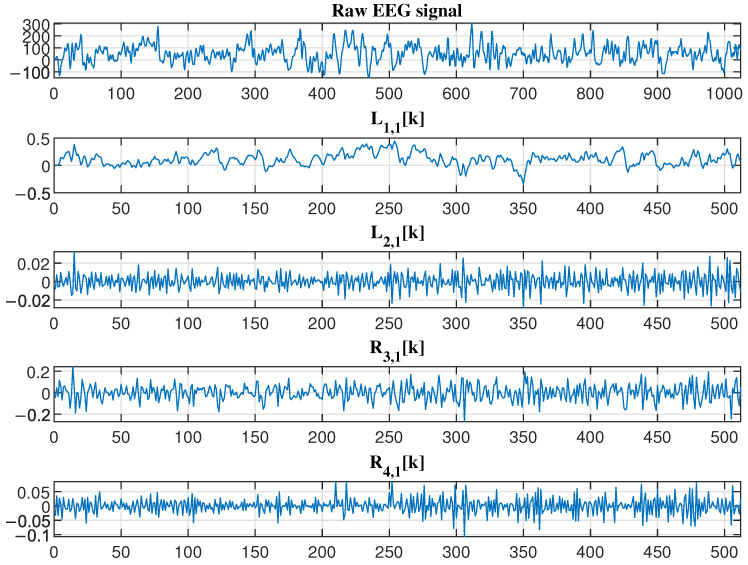
The raw EEG and, for each SSA component (i=1,2), the corresponding outputs of the Haar analysis filter bank: the low-frequency subband signal $L_{i,1}\left[k\right]$ and the high-frequency subband signal $R_{i,1}\left[k\right]$ of the first-layer analysis network.

For clarity in presentation, Figure 8 displays the 2 s EEG waveform segments along with their corresponding total two SSA components processed through the Haar analysis filter bank.

**Figure 9:**
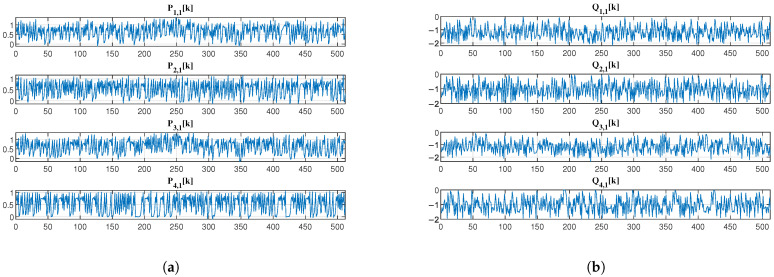
The output EEG (**a**) low-frequency subband signal $P_{i,1}\left[k\right]$ and (**b**) high-frequency subband signal $Q_{i,1}\left[k\right]$ of the first-layer analysis network.

**Figure 10:**
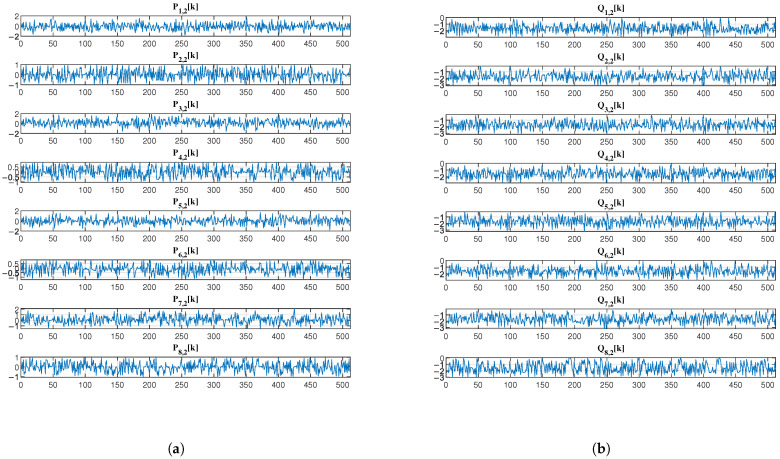
The output EEG (**a**) low-frequency subband signal $P_{i,2}\left[k\right]$ and (**b**) high-frequency subband signal $Q_{i,2}\left[k\right]$ of the second-layer analysis network.

Figure 9 and Figure 10 further illustrate the output signals of the first layer ($P_{i,1}\left[k\right]$ and $Q_{i,1}\left[k\right]$ for i=1,2,3,4) and the second layer ($P_{i,2}\left[k\right]$ and $Q_{i,2}\left[k\right]$ for i=1,2,…,8) of the analysis network, respectively. Visually, the encrypted EEG signals exhibit noise-like characteristics with apparent randomness. Compared to the method in [5], our system provides enhanced flexibility in decomposition components, enabling more versatile multi-party collaborative encryption. This is achieved through the hierarchical network structure that generates progressively more components at each layer while maintaining the desired security properties.

### 4.4. Security Analysis

We conduct a security assessment of the proposed encryption framework by evaluating nine distinct cryptographic approaches: the logistic chaotic filter bank approach [5], our SSA combined with 1-layer, 2-layer, and 3-layer chaotic networks, fully homomorphic encryption (FHE) [14], basic SMPC using simple Lagrange interpolation (Shamir’s scheme) [10], enhanced SMPC with finite field Lagrange interpolation (Robust BGW variant) [11,12], AES-based blockchain encryption, and K-sine-transform-based coupling chaotic system [6]. Our analysis employs three principal security metrics: power spectral density (PSD) analysis to evaluate frequency-domain whitening effects, power ratios analysis to quantify encrypted signal randomness, and correlation analysis assessing both plaintext–ciphertext relationships and inter-ciphertext dependencies.

#### 4.4.1. Power Spectral Density Analysis

**Figure 11:**
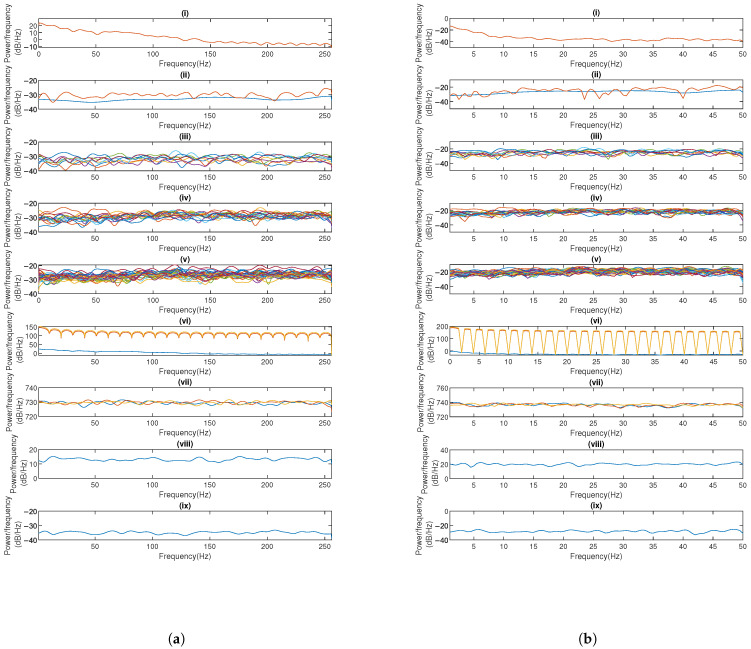
Encrypted signal’s PSD of (**a**) EEG and (**b**) PPG across eight encryption methods, with each subplot representing (i) the raw signal, (ii) the logistic chaotic filter bank approach from [5], (iii) our SSA with 1-layer chaotic network, (iv) our SSA with 2-layer network, (v) our SSA with 3-layer network, (vi) basic SMPC with simple Lagrange interpolation (Shamir’s scheme), (vii) enhanced SMPC with finite field Lagrange interpolation (Robust BGW variant), (viii) AES-based blockchain, and (ix) K-sine-transform-based coupling chaotic system.

PSD analysis reveals spectral differences between original and encrypted signals. An ideal encryption produces spectrally uniform outputs resembling white noise. As demonstrated in Figure 11a,b, while original EEG displays characteristic 0–50 Hz neural oscillations and PPG shows 0–5 Hz cardiac rhythms, all tested methods achieve relative spectral whitening, except Shamir’s scheme, which exhibits periodic fading and variations—a potential security weakness for frequency-domain cryptanalysis. In contrast, our 1–3-layer networks consistently generate relatively flat PSD profiles across the entire frequency spectrum (0–256 Hz for EEG, 0–50 Hz for PPG), effectively removing physiological signatures and inter-signal correlations. This spectral uniformity across network depth (1–3 layers) configurations demonstrates robust encryption performance. Given the nature of FHE, where users can only view the results of computations rather than directly accessing the ciphertext, security analyses typically do not encompass FHE.

#### 4.4.2. Power Ratio Analysis

**Figure 12:**
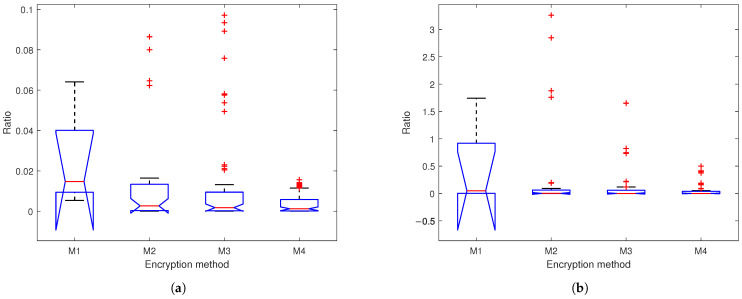
Box plots of ratios of (**a**) EEG and (**b**) PPG based on M1: chaotic filter bank in [5]; M2: our SSA with 1-layer chaotic network; M3: our SSA with 2-layer network; M4: our SSA with 3-layer chaotic network.

The power ratio metric quantifies the encryption strength by comparing the power of input subband signals to their corresponding chaotic encrypted outputs. Figure 12 presents box plots of the power ratios across four encryption methods, where the ratios are defined as $\frac{\sum_{\forall k}{\left|X_{i}\left[k\right]\right|}^{2}}{\sum_{\forall k}{\left|\left(Y_{i}\left[k\right]-X_{i}\left[k\right]\right)\right|}^{2}}$, (where *X* is input, *Y* is output, and i=1,2) for M1 and $\frac{\sum_{\forall k}{\left|L_{i,l}\left[k\right]\right|}^{2}}{\sum_{\forall k}{\left|\left(P_{i,l}\left[k\right]-L_{i,l}\left[k\right]\right)\right|}^{2}}$ or $\frac{\left(\sum_{\forall k}{\left|R_{i,l}\left[k\right]\right|}^{2}\right)}{\left(\sum_{\forall k}{\left|\left(Q_{i,l}\left[k\right]-R_{i,l}\left[k\right]\right)\right|}^{2}\right)}$ for M2–M4. The results demonstrate a progressively stronger suppression of signal residual energy across network depths, with median ratios approaching zero and distributions becoming tighter in deeper layers. This consistent reduction in power ratios confirms the enhanced security of our SSA with the multi-layer encryption method, significantly increasing the difficulty in recovering original information.

Table 2 further compares nine encryption methods, revealing that methods based on Shamir’s scheme and the robust BGW variant show complete signal suppression. Notably, methods based on AES and K-sine-transform exhibit higher absolute means (>0.48 for EEG, >1.0 for PPG).

#### 4.4.3. Correlation Analysis

A system is considered well-encrypted if the encrypted signals are highly uncorrelated with the original pre-encryption signals and if the different parts of the encrypted ciphertext block are also uncorrelated with each other. These properties prevent plaintext recovery from partial ciphertext and enforce collaborative decryption, where all parties’ ciphertexts are required for reconstruction.

The correlation coefficient of two signals is defined as

(18) $$r\left(X,Y\right)=\frac{1}{N}\sum_{i=1}^{N}\left(\frac{x_{i}-\bar{X}}{\sigma_{X}}\right)\left(\frac{y_{i}-\bar{Y}}{\sigma_{Y}}\right),$$

where $\bar{X}$ and $\bar{Y}$ are the average of these two signals, respectively, i.e., $\bar{X}=\frac{1}{N}\sum_{i=1}^{N}x_{i}$ and $\bar{Y}=\frac{1}{N}\sum_{i=1}^{N}y_{i}$, and $\sigma_{X}$ and $\sigma_{Y}$ are the variance of these two signals, respectively, i.e., $\sigma_{X}=\sqrt{\frac{\sum_{i=1}^{N}{\left(x_{i}-\bar{X}\right)}^{2}}{N}}$ and $\sigma_{Y}=\sqrt{\frac{\sum_{i=1}^{N}{\left(y_{i}-\bar{Y}\right)}^{2}}{N}}$.

**Figure 13:**
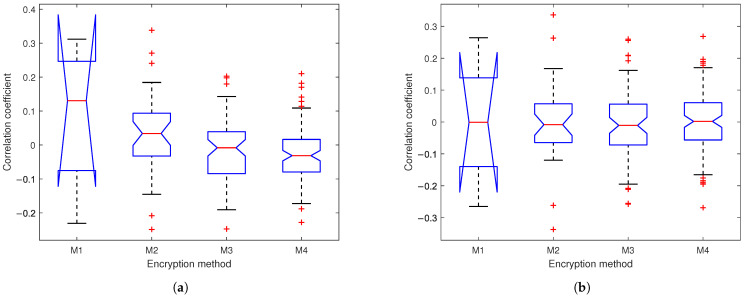
Box plots of correlation coefficients between inputs and outputs for (**a**) EEG and (**b**) PPG based on M1: chaotic filter bank in [5]; M2: our SSA with 1-layer chaotic network; M3: our SSA with 2-layer network; M4: our SSA with 3-layer chaotic network.

Figure 13 presents the correlation coefficients between inputs and outputs across four encryption methods (M1–M4). For M1, the correlation coefficients between the polyphase filter outputs and the chaotic encryption outputs. For M2–M4, the correlation coefficients between inputs $L_{i,l}\left[k\right]$, $R_{i,l}\left[k\right]$ and outputs $P_{i,l}\left[k\right]$, $Q_{i,l}\left[k\right]$ are calculated. Results show progressively lower correlations from M1 to M4 with medians approaching 0 and tighter coefficient distributions in deeper networks for both EEG and PPG.

Table 3 compares correlation coefficients for nine encryption methods on EEG/PPG signals. While some methods show slightly better decorrelation than our SSA-based chaotic network, our approach achieves sufficiently low correlations (<0.1) that decrease further with network depth (3-layer: EEG = 0.0545, PPG = 0.0641). All methods outperform Shamir’s scheme and [5], with EEG consistently showing better decorrelation than PPG.

Define the autocorrelation coefficient matrix of the $X_{i}$, for i=1,2,…,m as

(19) $$R_{a}=\left(\begin{array}{cccc} r\left(X_{1},X_{1}\right) & r\left(X_{1},X_{2}\right) & \ldots & r\left(X_{1},X_{m}\right)\\ r\left(X_{2},X_{1}\right) & r\left(X_{2},X_{2}\right) & \ldots & r\left(X_{2},X_{m}\right)\\ \vdots & \vdots & \ddots & \vdots \\ r\left(X_{m},X_{1}\right) & r\left(X_{m},X_{2}\right) & \ldots & r\left(X_{m},X_{m}\right) \end{array}\right),$$

and the intercorrelation coefficient matrix of the $X_{i}$ and $Y_{i}$, for i=1,2,…,m as

(20) $$R_{i}=\left(\begin{array}{cccc} r\left(X_{1},Y_{1}\right) & r\left(X_{1},Y_{2}\right) & \ldots & r\left(X_{1},Y_{m}\right)\\ r\left(X_{2},Y_{1}\right) & r\left(X_{2},Y_{2}\right) & \ldots & r\left(X_{2},Y_{m}\right)\\ \vdots & \vdots & \ddots & \vdots \\ r\left(X_{m},Y_{1}\right) & r\left(X_{m},Y_{2}\right) & \ldots & r\left(X_{m},Y_{m}\right) \end{array}\right).$$

**Figure 14:**
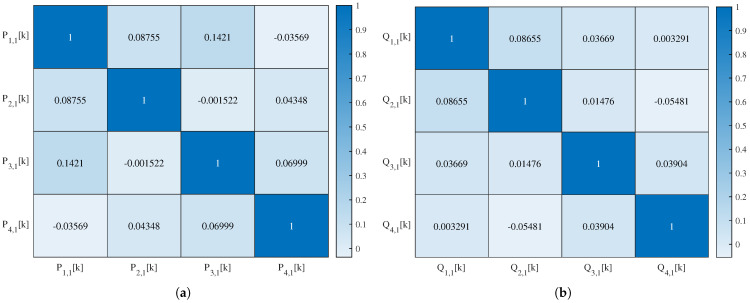
Heat map of the autocorrelation coefficient of (**a**) $P_{i,1}\left[k\right]$ and (**b**) $Q_{i,1}\left[k\right]$ of EEG encrypted by the 1-layer network.

**Figure 15:**
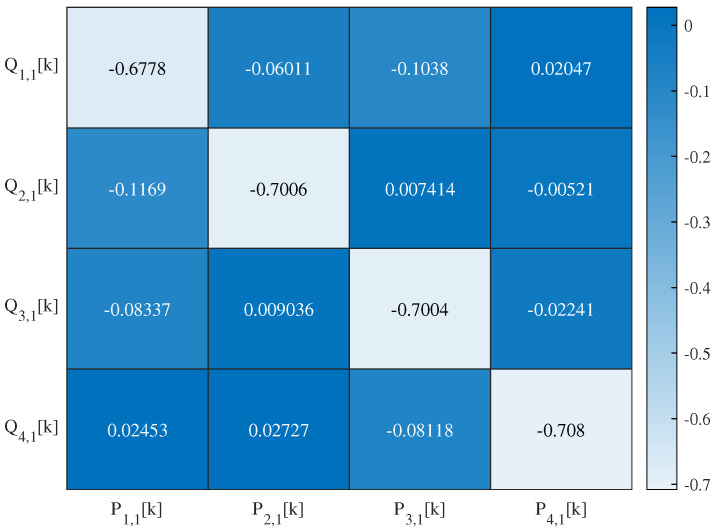
Heat map of the interrelation coefficient of EEG encrypted by the 1-layer network.

**Figure 16:**
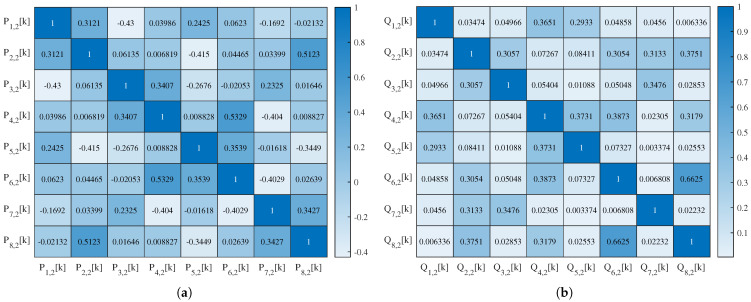
Heat map of the autocorrelation coefficient of (**a**) $P_{i,2}\left[k\right]$ and (**b**) $Q_{i,2}\left[k\right]$ of EEG encrypted by the 2-layer network.

**Figure 17:**
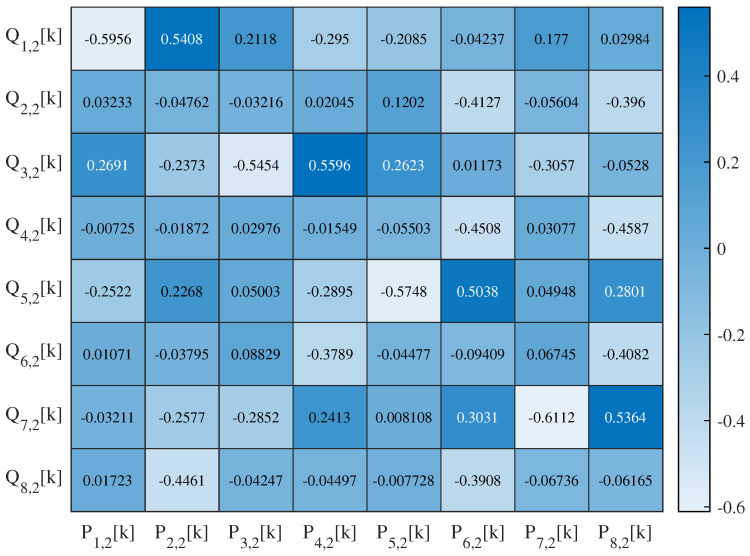
Heat map of the interrelation coefficient of EEG encrypted by the 2-layer network.

The 1-layer SSA-chaotic network produces four subband outputs, with their autocorrelation and interrelation matrices’ heat maps visualized in Figure 14 and Figure 15, respectively. For the 2-layer variant (eight outputs), the corresponding matrices’ heat maps are shown in Figure 16 and Figure 17. For conciseness, only the autocorrelation and interrelation matrices of the EEG encrypted by the 1-layer and 2-layer networks are presented here.

The other correlation coefficients for encrypted signals were classified as follows: weak ($|r\left(X,Y\right)|\;<0.3$), moderate ($0.3\leq |r\left(X,Y\right)|\;<0.7$), and strong ($|r\left(X,Y\right)|\;\geq 0.7$), based on established statistical conventions. For the 1-layer chaotic mapping network, most coefficients fell in the weak range, with fewer moderate (e.g., $|r\left(L_{2,1},L_{1,1}\right)|\;=0.50$) or strong (e.g., $|r\left(R_{3,1},L_{1,1}\right)|\;=0.89$) cases. For the 2-layer chaotic mapping network, coefficients are generally weak, with a few moderate or strong correlations.

**Figure 18:**
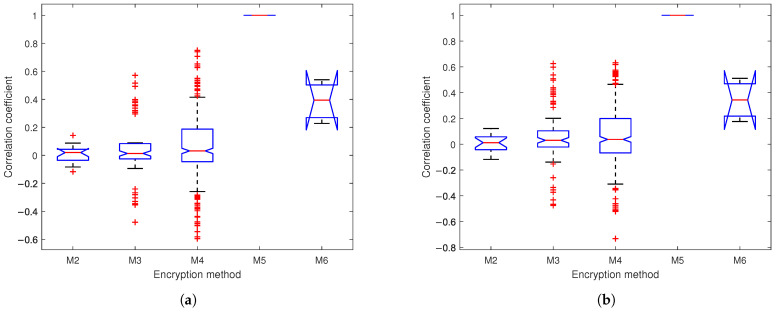
Box plots of correlation coefficients between encrypted signals for (**a**) EEG and (**b**) PPG based on M2: our SSA with 1-layer chaotic network; M3: our SSA with 2-layer network; M4: our SSA with 3-layer chaotic network; M5: Shamir’s scheme; M6: robust BGW variant.

Figure 18 presents box plot comparisons of encrypted signals’ intercorrelations for EEG and PPG across five multi-party encryption methods. Our approach consistently outperforms the other two comparison methods across network depths (1-layer to 3-layer), with 1-layer correlations <0.1 (excluding outliers)—significantly lower than M4 (>0.2) and M5 (≈1 median). While correlations increase slightly with deeper networks, our method relatively outperforms alternatives, demonstrating robust security against correlation attacks while preserving multi-layer practicality for multi-party systems where threshold decryption is required.

### 4.5. Verification Performance

Our experimental results validate the verification mechanism described in Section 2.3, where the system’s redundant architecture enables two independent decryption paths to converge to identical decrypted outputs with remarkable precision. Both the first and second synthesis network layer demonstrate near-perfect decryption consistency, as shown in Table 4 and Table 5, respectively. The paired decryption paths achieve MSE on the order of $10^{-32}$ and PRD between $10^{-15}$ and $10^{-10}$, with all observed deviations attributable to floating-point arithmetic limitations. The consistent output matching enables tamper detection through deviation monitoring and cryptographic integrity verification without external reference signals.

### 4.6. Decryption Error Analysis Under Collaborator Tampering Scenarios

We modified the encrypted signal by replacing its first data point with a uniform random value. Although theory predicts near-perfect consistency across synthesis layers and final reconstruction, our experimental results (Table 6) reveal significant discrepancies. Quantitative evaluation using MSE and PRD metrics demonstrates that even minimal ciphertext alterations (as small as 0.2%) produce clearly detectable tampering artifacts in the decrypted output.

### 4.7. Encryption and Decryption Efficiency Analysis

During secure channel transmission, the time spent in the encryption and decryption directly impacts data exchange performance. The EEG with a duration of 2 s and the PPG with a duration of 5 s are selected. We test the actual encryption time on a computer with 12th Gen Intel(R) Core(TM) i9-12900K 3.20 GHz processor. The encryption and decryption times are calculated separately for comparison, as shown in Table 7. The results demonstrate that our method achieves millisecond encryption/decryption latency for signal processing, fulfilling the low-latency demands of telemedicine. Morever, the increase in the number of layers does not significantly increase the time required for encryption and decryption. This is probably because the CPU’s processes on the computer cannot be shut down.

### 4.8. Performance Across Window Lengths

**Figure 19:**
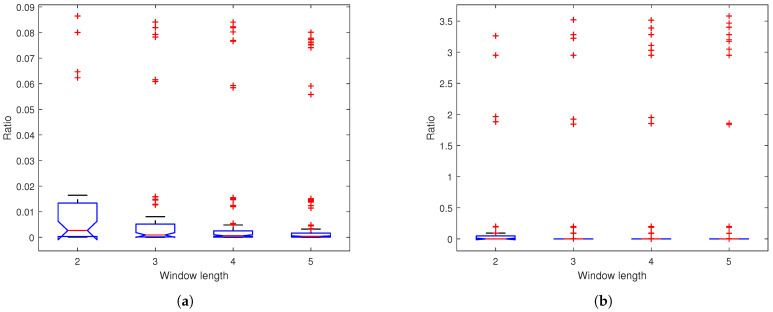
Box plots of ratios for (**a**) EEG and (**b**) PPG based on our SSA with 1-layer chaotic network (window length: 2–5).

**Figure 20:**
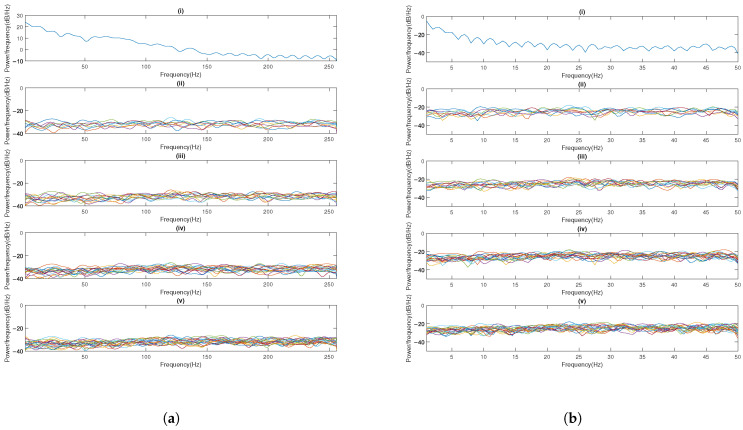
Encrypted signal’s PSD of (**a**) EEG and (**b**) PPG based on our SSA with 1-layer chaotic network, with each subplot representing (i) original, (ii) the SSA window length of 2, (iii) the SSA window length of 3, (iv) the SSA window length of 4, and (v) the SSA window length of 5.

In this example, we utilize a 1-layer network. Figure 19 displays the box plot of the power ratio for varying SSA window lengths, indicating that, as the window length increases, the distribution range of the ratio diminishes. Table 8 presents the absolute means and variances of correlation coefficients between encrypted and original signals across varying window lengths. The results demonstrate an inverse relationship between window length and correlation coefficients: as window length increases, both the absolute mean and variance of the correlation coefficients decrease. While longer window length reduces correlation coefficients, it also increases computational costs from SVD. Therefore, optimal window lengths should balance these factors. Figure 20 presents the PSD of encrypted signals across varying window lengths. The results show consistently flat frequency spectra, indicating stable encryption performance regardless of window length.

## 5. Conclusions

This paper presents a verifiable multi-party encryption system for biosensor signals using SSA-based chaotic filter bank networks, demonstrating three key advances: (1) adaptive SSA decomposition with window-length-optimized component extraction for non-stationary EEG and PPG, (2) enhanced chaotic performance through multi-layer chaotic filter bank network diffusion, and (3) integrated consistency verification for collaborative decryption. The computer simulation results demonstrate that the proposed encryption system exhibits robust performance across enhanced key complexity, with the security validated and improved computational efficiency.

However, SMPC retains inherent limitations that our approach will overcome through flexible storage–communication tradeoffs in future deployments, specifically, (1) ciphertexts will remain opaque to all participants while enabling multi-party decryption through distributed key sharing, eliminating SMPC’s massive communication overhead at the cost of increased local storage demands; and (2) planned hardware-aware SSA optimizations will address real-time processing efficiency for clinical biosensor applications.

## Figures and Tables

**Table 1:** The reconstruction performance metrics (MSE and PRD) based on the logistic chaotic filter bank in [5], our SSA with 1-layer chaotic network, our SSA with 2-layer chaotic network, and our SSA with 3-layer chaotic network.

Signal	EEG		PPG	
**Encryption Methods**	**MSE**	**PRD**	**MSE**	**PRD**
Logistic chaotic filter bank of [5]	$1.6335\times 10^{-27}$	$3.7036\times 10^{-14}$	$5.4749\times 10^{-27}$	$1.6972\times 10^{-14}$
Our SSA with 1-layer chaotic network	$3.6665\times 10^{-27}$	$5.5487\times 10^{-14}$	$9.2309\times 10^{-27}$	$2.2038\times 10^{-14}$
Our SSA with 2-layer chaotic network	$1.2784\times 10^{-26}$	$1.0361\times 10^{-13}$	$1.6074\times 10^{-26}$	$2.9081\times 10^{-14}$
Our SSA with 3-layer chaotic network	$1.7115\times 10^{-26}$	$1.1988\times 10^{-13}$	$2.9525\times 10^{-26}$	$3.9413\times 10^{-14}$

**Table 2:** The absolute means and variances of power ratios for 2 s EEG and 5 s PPG accross nine encryption methods.

Signal	EEG		PPG	
**Encryption Methods**	**Absolute Mean**	**Variance**	**Absolute Mean**	**Variance**
Logistic chaotic filter bank in [5]	0.0248	0.0005	0.4610	0.5483
Our SSA with 1-layer chaotic network	0.0133	0.0006	0.3255	0.6895
Our SSA with 2-layer chaotic network	0.0124	0.0006	0.1214	0.1095
Our SSA with 3-layer chaotic network	0.0039	0.0000	0.0479	0.0096
FHE ^1^	−	−	−	−
Shamir’s scheme	0.0000	0.0000	0.0000	0.0000
Robust BGW variant	0.0000	0.0000	0.0000	0.0000
AES-based blockchain ^2^	0.4836	−	1.8669	−
K-sine-transform-based coupling chaotic system ^3^	1.0085	−	1.0023	−

^1^ The methods based on FHE was excluded from absolute mean and variance calculations due to the ciphertext cannot be obtained. ^2,3^ The methods based on AES-based blockchain and K-sine-transform were excluded from variance calculations because they do not support multi-party encryption.

**Table 3:** The absolute means and variances of the correlation coefficients for 2 s EEG and 5 s PPG accross nine encryption methods.

Signal	EEG		PPG	
**Encryption Methods**	**Absolute Mean**	**Variance**	**Absolute Mean**	**Variance**
Logistic chaotic filter bank in [5]	0.2010	0.0401	0.1393	0.0351
Our SSA with 1-layer chaotic network	0.0982	0.0156	0.0850	0.0154
Our SSA with 2-layer chaotic network	0.0756	0.0092	0.0847	0.0125
Our SSA with 3-layer chaotic network	0.0545	0.0046	0.0641	0.0069
FHE	−	−	−	−
Shamir’s scheme	1	0	0.9589	0
Robust BGW variant	0.0402	0.0014	0.0490	0.0008
AES-based blockchain	0.0263	−	0.0285	−
K-sine-transform-based coupling chaotic system	0.0264	−	0.0607	−

**Table 4:** Matching MSE and PRD of paired decryption outputs for EEG and PPG in the first synthesis network layer.

Signal	EEG		PPG	
**Paired Decryption Results**	**MSE**	**PRD**	**MSE**	**PRD**
$L_{1,1}^{\prime }\left[k\right]$	$4.3886\times 10^{-32}$	$1.3091\times 10^{-13}$	$8.2765\times 10^{-32}$	$3.2995\times 10^{-14}$
$L_{2,1}^{\prime }\left[k\right]$	$3.3727\times 10^{-32}$	$2.0994\times 10^{-12}$	$3.9067\times 10^{-32}$	$3.4297\times 10^{-10}$
$R_{1,1}^{\prime }\left[k\right]$	$5.4613\times 10^{-32}$	$3.1783\times 10^{-13}$	$8.5657\times 10^{-32}$	$9.2950\times 10^{-13}$
$R_{2,1}^{\prime }\left[k\right]$	$1.0464\times 10^{-32}$	$1.1596\times 10^{-12}$	$7.0762\times 10^{-32}$	$3.9852\times 10^{-11}$

**Table 5:** Matching MSE and PRD of paired decryption outputs for EEG and PPG in the second synthesis network layer.

Signal	EEG		PPG	
**Paired Decryption Results**	**MSE**	**PRD**	**MSE**	**PRD**
$L_{1,2}^{\prime }\left[k\right]$	$2.3699\times 10^{-32}$	$2.0693\times 10^{-14}$	$4.5569\times 10^{-32}$	$1.4695\times 10^{-14}$
$L_{2,2}^{\prime }\left[k\right]$	$2.2611\times 10^{-32}$	$2.3431\times 10^{-14}$	$2.4928\times 10^{-32}$	$2.4944\times 10^{-14}$
$L_{3,2}^{\prime }\left[k\right]$	$2.6543\times 10^{-32}$	$1.1282\times 10^{-14}$	$4.4123\times 10^{-32}$	$1.4652\times 10^{-14}$
$L_{4,2}^{\prime }\left[k\right]$	$2.1918\times 10^{-32}$	$2.2143\times 10^{-14}$	$2.1516\times 10^{-32}$	$2.3808\times 10^{-14}$
$R_{1,2}^{\prime }\left[k\right]$	$2.3678\times 10^{-32}$	$1.2006\times 10^{-14}$	$2.7943\times 10^{-32}$	$8.3029\times 10^{-15}$
$R_{2,2}^{\prime }\left[k\right]$	$2.0284\times 10^{-32}$	$1.2224\times 10^{-14}$	$3.1065\times 10^{-32}$	$1.5161\times 10^{-14}$
$R_{3,2}^{\prime }\left[k\right]$	$1.8907\times 10^{-32}$	$1.0872\times 10^{-14}$	$2.9558\times 10^{-32}$	$8.7092\times 10^{-15}$
$R_{4,2}^{\prime }\left[k\right]$	$2.1714\times 10^{-32}$	$1.2914\times 10^{-14}$	$2.9434\times 10^{-32}$	$1.5228\times 10^{-14}$

**Table 6:** Matching MSE and PRD of paired decryption outputs for EEG and PPG in 2-layer synthesis network.

Signal	EEG		PPG	
**Paired Decryption Results**	**MSE**	**PRD**	**MSE**	**PRD**
$x^{\prime }\left[k\right]$	19.7632	5.4167	19.5551	1.0225
$L_{error,1}^{\prime }\left[k\right]$ or $R_{error,1}^{\prime }\left[k\right]$	0.0013	43.0444	0.0012	47.6284
$L_{error,2}^{\prime }\left[k\right]$ or $R_{error,2}^{\prime }\left[k\right]$	0.0013	3.0426	0.0012	3.0111

**Table 7:** Encryption time and decryption time of EEG and PPG.

Signal	EEG		PPG	
**Encryption Methods**	**Encryption Time (s)**	**Decryption Time (s)**	**Encryption Time (s)**	**Decryption Time (s)**
Logistic chaotic filter bank in [5]	0.0017	0.0017	0.0016	0.0018
Our SSA with 1-layer chaotic network	0.0078	0.0050	0.0083	0.0025
Our SSA with 2-layer chaotic network	0.0098	0.0066	0.0063	0.0015
Our SSA with 3-layer chaotic network	0.0086	0.0011	0.0098	0.0067
FHE	0.1132	0.0196	0.0966	0.0125
Shamir’s scheme	0.0002	0.0001	0.0002	0.0001
Robust BGW variant	0.0023	0.0048	0.0012	0.0017
AES-based blockchain	0.0005	0.0001	0.0006	0.0001
K-sine-transform-based coupling chaotic system	0.0142	0.0092	0.0079	0.0078

**Table 8:** The absolute mean and variance of the correlation coefficients between encrypted and original signals based on our SSA with 1-layer chaotic network (window length: 2–5).

Signal	EEG		PPG	
**Window Length**	**Absolute Mean**	**Variance**	**Absolute Mean**	**Variance**
2	0.0982	0.0401	0.0850	0.0154
3	0.0633	0.0074	0.0726	0.0108
4	0.0543	0.0055	0.0598	0.0075
5	0.0480	0.0043	0.0606	0.0065

## Data Availability

Data are contained within the article.
